# Study protocol for a multi‐centre parallel two‐group randomized controlled trial evaluating the effectiveness and impact of a pain assessment and management program for respite workers supporting children with disabilities

**DOI:** 10.1002/pne2.12014

**Published:** 2020-04-26

**Authors:** Lara M. Genik, C. Meghan McMurtry, Paula C. Barata, Chantel C. Barney, Stephen P. Lewis

**Affiliations:** ^1^ Department of Psychology University of Guelph Guelph ON Canada; ^2^ Pediatric Chronic Pain Program McMaster Children’s Hospital Hamilton ON Canada; ^3^ Children’s Health Research Institute London Canada; ^4^ Department of Pediatrics Western University London ON Canada; ^5^ Gillette Children’s Specialty Health Care Department of Educational Psychology University of Minnesota Minneapolis MN USA

**Keywords:** children, disabilities, education, pain, respite workers

## Abstract

**Objective:**

Pain is common and complex for children with intellectual and developmental disabilities (I/DD). Secondary caregivers such as respite workers are lacking important pain‐related information which can impact care. Here, we outline a randomized controlled trial (RCT) protocol testing the effectiveness of a pain training for respite workers supporting children with I/DD.

**Methods/design:**

Organizations enrolled in the RCT were randomly assigned to receive a 3‐3.5 hours pain or family‐centered care training. Data were collected immediately before, after, and 4‐6 weeks following completion of the training. Outcomes are as follows: pain knowledge (primary), pain assessment and management perceptions (secondary), training evaluations (secondary), and use of pain assessment and management skills (tertiary). Both quantitative and qualitative methodologies are being used including questionnaires, rating scales, a standardized vignette, and focus groups.

**Conclusions:**

Results from this trial will be used to further understand the impact of the pain training and inform next steps related to implementation. TRIAL REGISTRATION: ClinicalTrials.gov identifier: NCT03421795.

## 1 INTRODUCTION

Pain is common for children with intellectual and developmental disabilities (I/DD)^1^; limited communication skills and differences in pain expression also make assessment and management challenging.^2^, ^3^ There are short‐ and long‐term consequences of inadequately managed pain in those with I/DD including disrupted sleep and reduced adaptive functioning in communication, daily living skills, socialization, and motor skills.^4^, ^5^ There has been progress in assessing pain of children with I/DD using structured behavioral measures such as the *Non‐Communicating Children's Pain Checklist—Revised* ^6^ and self‐report adaptations [eg, 7]. Some work has also been conducted on pain management for children with I/DD. Existing research has alluded to pharmacological, physical, psychological, and process‐related management strategies as important and helpful in a disability context [eg, forms of distraction in a postoperative context: 7, 8].

To date, most work related to pain in children with I/DD has focused on primary caregivers and healthcare providers [eg, 2, 6, 9]. However, secondary caregivers such as respite workers (RW) also spend considerable time with children with I/DD. Initial research addressing this knowledge gap demonstrated that RW who frequently spend time with these children may hold inaccurate beliefs about pain and do not typically have access to specialized pain education related to children with I/DD.^10^ Recent work in related areas (eg, residential support workers of adults with I/DD, school nurses for children with I/DD) has illuminated similar challenges including: inaccurate beliefs,^11^ lack of knowledge,^11^, ^12^ and role confusion with other support staff.^12^ Access to relevant pain knowledge is critical so caregivers of children with I/DD can provide appropriate care.

Recognizing the knowledge gap in the children's respite community, Genik, and colleagues^13^ conducted a two‐phase study which first gathered information about RW’s pain‐related experiences and perceived training needs and preferences. This information was used in tandem with extant literature to develop a relevant, empirically informed pain assessment and management training, called *Let's Talk About Pain*, for RW who support children with I/DD. Pilot study results demonstrated (a) increased pain‐related knowledge, (b) increased self‐reported perceptions of the feasibility of and participants’ perceived confidence and skill in pain assessment and management (herein “pain assessment and management perceptions”), and (c) favorable endorsement of the training by RW.^13^ Identified next steps were to systematically evaluate the training's impact on both knowledge and skill use with a larger, more diverse sample and have a longer‐term follow‐up.

This paper presents the complete randomized controlled trial (RCT) protocol used to test the effectiveness and impact of *Let's Talk About Pain* on RW’s pain‐related knowledge, beliefs, and assessment and management approaches when caring for children with I/DD. This paper has been published in advance of the RCT study results to ensure that enough detail regarding the RCT and its development can be available to researchers and clinicians, both of whom could benefit from the information. For example, this information could be important for future RCT implementation, study replication, and application in clinical settings. The *Standard Protocol Items: Recommendations for Interventional Trials (SPIRIT) 2013* ^14^ guideline for the minimum content which should be reported for trial protocols was used to guide protocol content within this manuscript. Additional information not included in the current manuscript is available upon request (eg, copies of consent forms).

## 2 TRIAL STATUS

This trial is registered with clinicaltrials.gov (identifier: NCT03421795). Data collection was completed in August 2018; analyses are ongoing. Researchers have no conflicts of interest.

## 3 OUTCOME MEASURES AND HYPOTHESES

Participant outcome measures were as follows: (a) pain‐related knowledge [primary], (b) pain assessment and management perceptions [secondary], (c) participants’ training endorsements [secondary], and (d) use of evidence‐based pain assessment and management strategies [tertiary]. Between‐group hypotheses predicted that immediately post and at 4‐ to 6‐week follow‐up, participants receiving the pain training would demonstrate significantly higher pain knowledge and pain assessment and management perceptions compared with the control group. It was also predicted that at follow‐up participants receiving the pain training would have significantly higher levels of evidence‐based pain assessment and management strategy use compared with the control group. Within‐group hypotheses for those receiving the pain training predicted significant increases on pain knowledge and pain assessment and management perceptions from pre to post, with maintenance of these gains from post to follow‐up. It was also hypothesized that participants would provide favorable endorsements of the training program. Finally, significant increases from pre to follow‐up for evidence‐based pain assessment and management strategy use were hypothesized. Results from the RCT are to be published in two separate manuscripts: one reviewing the impact of the training on participant knowledge and perceptions, and the other reviewing participants’ training endorsements and its impact on their strategy use in the workplace.

## 4 DESIGN/METHODS

### 4.1 Study design and procedure

**FIGURE 1:**
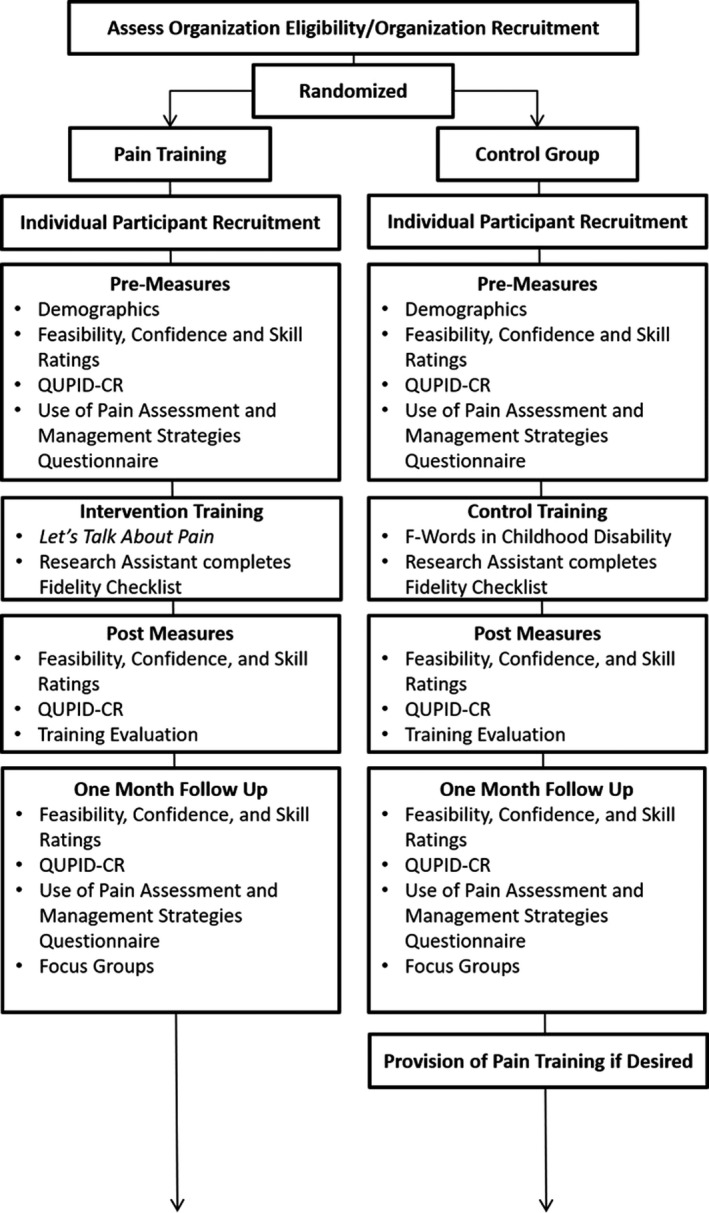
Outline of study procedures and methodology used for both groups of participants

The RCT represents a multi‐center parallel two group (pain training, control training) design. Data were collected in‐person using hard copy questionnaires immediately before and after an initial training, and again at a 4‐ to 6‐week follow‐up time point using hard copy questionnaires and focus group methodology (See Figure 1 for procedure). Participating organizations were given information about the general purpose of the study as well as the training topic(s), but were not made explicitly aware of whether they were allocated to the control or intervention condition. Participants were told that the purpose of the study was to learn about the impact that training can have on RW knowledge about caring for children with I/DD. No information was provided to participants about study hypotheses.

### 4.2 Organization recruitment

Ethical approval was secured from the University research ethics board. Participant recruitment occurred in collaboration with children's respite organizations in Ontario. A researcher‐compiled organization database of 95 potentially eligible organizations developed from (a) a list of respite organizations in Ontario from www.respiteservices.com, and (b) online searches for children's respite services in Ontario was used. These organizations were approached by telephone and/or email. Organizations in closest proximity to the researchers’ home city were contacted first with organizations further away contacted as needed to achieve optimal sample size.

### 4.3 Randomization

As organizations agreed to host the program(s) for their RW, the entire organization was randomly assigned to either the control or intervention group. This means that all participating staff from a given organization participated in the same condition. Sequentially numbered, sealed, opaque envelopes (SNOSE) with a 1:1 allocation ratio were created by a research assistant not involved in study recruitment, group allocations, training, or data collection processes. Once assigned, primary investigators were no longer blind to the group allocation.

### 4.4 Participant recruitment

Researchers recruited and sought written informed consent from participants at each initial training hosted by participating organizations. Participants had to be proficient in the English language and at least 18 years of age and employed through participating organizations as RW supporting children with I/DD in any setting (eg, family home, community, group homes) in order to participate. Ineligible staff or those who choose not to participate in the study component could still attend the training(s).

### 4.5 Sample size

G*Power 3^15^ was used to conduct a power analysis based on our previous *Let's Talk About Pain* training pilot study.^13^ Based on the smallest effect size (*d* = 0.90 [effect size *f* = 0.45]; original range of effect sizes *d* = 0.90‐1.71), a very small sample size (16) was required at power of 0.95 and alpha of 0.05 to detect an overall interaction effect of group by time. A much more conservative effect size (*d* = 0.25 [effect size *f* = 0.1250]) was also used to calculate the most stringent estimate at power of 0.95 and alpha of 0.05, resulting in a total target sample size of approximately 84 participants per group (168 participants total; [15). We aimed for our study sample size to fall in the higher end of this range.

## 5 INTERVENTIONS

### 5.1 General training characteristics

Each training occurred in‐person with no more than 30 participants, lasting 3‐3.5 hours with one or two breaks totaling approximately 30 minutes. Training sessions were interactive in nature (eg, group discussions, case studies) and structured with a Power‐Point presentation with notes and provision of relevant resources. A standardized fidelity checklist was used to document key points covered (or not covered in the control group) during the training.

### 5.2 Let's talk about pain training

Previously developed and piloted by Genik and colleagues,^13^ the training's content was specific to children with I/DD and focused broadly on providing information on what pain is, pain expression, pain assessment, and pain management specific to a respite context; detailed training outline is available upon request. *Let's Talk About Pain* is empirically informed and covers all relevant aspects of Chapter 43 of the IASP Core Curriculum for Professional Education in Pain.^16^ The training was facilitated by the same facilitator throughout the study.

### 5.3 Control training

Participants in the control group completed a training about a family‐centered care approach. Specifically, the training provided information about the F‐words of childhood disability (function, family, fitness, fun, friends, future; [17]) and how to implement this framework in a respite setting. This translational work is based on the World Health Organization's (2001) International Classification of Functioning, Disability and Health Framework. The topic is fitting given its relevance to care of children in respite settings and its lack of focus on pain. This training was facilitated by a PhD Candidate from McMaster University (Hamilton, ON, Canada).

## 6 OUTCOME MEASURES

### 6.1 Primary

Participants’ pain‐related knowledge was assessed at pre, post, and follow‐up using the *Questionnaire for Understanding Pain in Individuals with Intellectual Disabilities—Caregiver Version Revised (QUPID‐CR)*, a 39‐item true/false and multiple‐choice questionnaire. Single points are awarded for each correct response to compute a total score out of 39, and higher scores represent greater levels of knowledge. The QUPID‐CR was developed following initial responsivity and item‐level analyses of the original QUPID‐C^18^ completed prior to use in this RCT.^19^ Like the QUPID‐C, the QUPID‐CR is based on existing literature and the International Association for the Study of Pain's (IASP) core curriculum (Chapter 43; [16]).

### 6.2 Secondary

Participants provided 6 self‐report ratings (Pain Assessment and Management Perceptions) of their perceptions of (1) the feasibility of (0 = *Not Feasible At All,* 10 = *Highly/Extremely Feasible*), (2) their confidence in (0 = *Not Confident At All*, 10 = *Highly/Extremely Confident*), and (3) their skill in (0 = *Not Skilled At All*, 10 = *Highly/Extremely Skilled*) pain assessment and management for children with I/DD at all three time points. These ratings have shown responsivity to training.^13^, ^18^

Participants also completed a researcher‐developed questionnaire at post, rating their level of agreement (0 = *strongly disagree* to 10 = *strongly agree*) with different statements about the training program (eg, effectiveness of format). Here, participants also provided responses to open‐ended questions related to the training program (eg, what component of the training they thought contributed most to their learning). This questionnaire is based on that which was used in the pilot study for the *Let's Talk About Pain* training.^13^

### 6.3 Tertiary

Participants’ use of pain assessment and management skills was assessed using both questionnaire and focus group methodology (see Figure 1). The *Use of Pain Assessment and Management Strategies Questionnaire* is a researcher‐generated questionnaire which includes open and closed questions about the following: (a) participants’ work in a respite setting (eg, number of shifts/hours per week) and (b) frequency of and types of pain assessment and management strategies used. These questions are intended to explore participants’ use of pain assessment and management strategies within the context of their work environment. This questionnaire also contains a previously developed vignette to explore their use of pain assessment and management strategies in a more standardized way.^20^ This vignette has shown divergent and convergent validity when compared to other pain‐related vignettes with different pain sources and background information.^20^

The aim of the focus group component was to explore the types of pain assessment, and management strategies RW have used on the job more in depth. Each began with a brief introduction of the purpose followed by semi‐structured questions about participants’: (1) opinions and knowledge about pain in children with I/DD and (2) experiences with pain assessment and management in respite settings, specifically in the time period between completion of the training and the follow‐up data collection. They were also asked about times when they remembered something from the training and whether they were able or unable to use it; the full focus group guide is available upon request. Audio‐recorded focus groups were 30 minutes to one hour in length, with no more than 12 participants per focus group.^21^ The number of focus groups was dependent on the number of participants attending each organization's follow‐up data collection. Focus groups were facilitated by the lead researcher with an accompanying research assistant taking field notes.^21^

## 7 PARTICIPANT COMPLIANCE AND LOSS TO FOLLOW‐UP

Given the single time point nature of the intervention and previous data from the *Let's Talk About Pain* pilot study,^13^ participant compliance was not of great concern. In contrast, the potential to lose participants at the follow‐up time point was more unknown. Incentives were used during the initial data collection (pre, post) and follow‐up to try to maximize participant retention. Specifically, participants received an entry into a $20 gift card draw (odds of winning: 1 in 25) and refreshments at each visit (ie, at the pre/post data collection date, and at follow‐up), a notebook and pen set at the initial time point, and a certificate along with a $20 honorarium at follow‐up.

## 8 ANALYSES

Research assistants double enter the data into an SPSS statistical analysis program which is stored on an encrypted, password protected e‐drive. Hard copy data will be stored for seven years after publication of results.

Both intention‐to‐treat (ITT) and per protocol (PP) analysis approaches will be used to analyze the data related to the study's primary and secondary outcomes regarding participants’ pain knowledge and pain assessment and management perceptions; ITT will be primary, and PP will be complementary.^22^, ^23^ After confirming there are no baseline differences between groups, seven 2 × 3 mixed analysis of variances (ANOVA) will be conducted. If statistical assumptions for these analyses are not met, a more conservative approach using bootstrapping will be considered and conducted if the program allows; SPPS has some limitations in this regard.^24^ The dependent variables for the different mixed ANOVAs will be the mean of participants’ pain‐related knowledge (primary outcome measure) and pain assessment and management perception rating scores (secondary outcome measures). In all seven analyses, the within‐subjects factor will be represented by participants’ scores on the related measure across pre, post, and follow‐up time periods, and the between‐subjects factor will be the condition (pain training or control training). In keeping with the study's specific hypotheses, follow‐up analyses using one‐way ANOVAs and paired samples *t* tests will be used as needed to further investigate differences in the dependent variables over time within the pain training group if significant group differences are found.

Frequency and descriptive analyses will be used to analyze participant open‐ended responses on the *Use of Pain Assessment and Management Strategies* and training evaluation questionnaire. To describe responses to open‐ended questions, coding schemes will be developed from an essentialist/realist perspective using an unconstrained matrix.^25^, ^26^ In this scheme, both inductive (ie, consideration of participant responses) and deductive (ie, consideration of relevant research literature) approaches will be incorporated as relevant. For example, category names and descriptions will reflect what is known from previous literature as relevant, but new categories will also be developed that may not be consistent with the literature should they arise. First, the primary investigator will become familiarized with the data, and initial categories will be generated and grouped into broader categories with definitions.^27^ These schemes will then be reviewed by additional researchers on the team who will: (a) help to ensure that the schemes are representative of the data and (b) further develop category definitions and examples.^27^ Following coding scheme development, two research assistants will be trained on these schemes. Participant responses will then be coded, and inter‐rater reliability for coding will be calculated using Cohen's Kappa. An inductive and essentialist/realist qualitative thematic analysis following the steps outlined by Braun and Clarke^28^ will be used to analyze focus group transcript data. Following familiarization with the data, transcriptions will be uploaded to NVivo12 software where meta‐ and subthemes will be derived, defined, and refined. How often a topic is raised, the length of time it is discussed, existing research literature, and applied researcher experience in the field will all be considered in developing these themes. Analyses will occur at the semantic level. Intervention and control focus groups will be analyzed separately from one another.

## 9 DISCUSSION

Pain is often underdiagnosed and treated for children with I/DD, and challenges with verbal communication and behavioral expression can further complicate pain assessment and management.^1^, ^3^ Secondary caregivers such as RW often spend time with children with I/DD yet are lacking important information about pain.^10^ Preliminary work suggests that pain‐related education can improve these caregivers’ knowledge and perceptions.^17^ These findings are well aligned with similar studies targeting pain assessment strategies for school nurses and residential support workers.^29^, ^30^

It is believed that the current project will provide more information about the impact of the *Let's Talk About Pain* training on participants’ knowledge and perceptions both in the short and longer term across organizations. Results from the RCT will also provide preliminary data on the impact this program may have on RW application of skills learned. If found to be effective, this RCT may provide researchers with further direction regarding implementation of the program across respite organizations, and next steps to encourage training uptake and skill application. The described RCT will be an important contribution to the literature on best practices in caring for children with I/DD.
